# Sodium content in commonly consumed away-from-home food in three areas of Metropolitan Lima, Peru

**DOI:** 10.17843/rpmesp.2023.403.12939

**Published:** 2023-09-27

**Authors:** Mayra Meza-Hernández, Rafael Durán-Galdo, Daniella Torres-Schiaffino, Lorena Saavedra-Garcia

**Affiliations:** 1 CRONICAS Centro de excelencia en enfermedades crónicas, Universidad Peruana Cayetano Heredia. Lima, Peru. Universidad Peruana Cayetano Heredia CRONICAS Centro de excelencia en enfermedades crónicas Universidad Peruana Cayetano Heredia Lima Peru

**Keywords:** Street food, Sodium, Fast Foods, Peru

## Abstract

The aim of this study was to characterize the sodium content in commonly consumed away-from-home food in three areas of Metropolitan Lima. We conducted a cross-sectional study, in which twenty frequently consumed foods were identified according to the place of sale. Sodium content was determined through atomic absorption spectroscopy in preparations collected in 2019. The median sodium content in street food products was 492.36 mg/100g (IQR: 83.93 - 918.78), 471.37 mg/100 g in artisanal food (IQR: 76.04 - 765.39) and 471.06 mg/100 g in fast food (IQR: 115.31 - 695.18). Sixty-five percent of the foods were classified as having high sodium content according to Peruvian regulations, while 30% of the preparations had high sodium content, according to UK parameters. Most food sold and consumed away from home have high sodium content. It is essential to engage all stakeholders involved in food preparation for away-from-home consumption in order to raise awareness and involve them in the promotion of policies aimed at reducing sodium intake.

## INTRODUCTION

In many countries, dietary sodium intake exceeds the World Health Organization (WHO) recommendation of 2000 mg of sodium per day, equivalent to 5000 mg of salt ^1^. This happens, for example, in Latin America, where the average sodium intake is 4130 mg per day ^2^. High sodium intake is one of the risk factors for the development of cardiovascular disease, renal failure and premature death ^1^.

Both processed and ultra-processed foods, as well as foods prepared outside the home, represent the main source of sodium intake in developed countries ^(^^3^. In contrast, the main source of sodium in developing countries is the salt added during home cooking ^4^. However, as part of the nutritional transition, the consumption of away-from-home food is increasing. Therefore, away-from-home food is currently of great interest because its consumption is very frequent and part of the gastronomic culture of these countries ^5^.

Several strategies have been developed in response to the increase in sodium consumption and its consequences on health. For example, Peru enacted Law No. 30021, “Law for the promotion of healthy eating for children and adolescents”, which establishes parameters for determining the high content of nutrients such as sodium in processed and ultra-processed foods ^6^. The United Kingdom has also established parameters for classifying processed and ultra-processed foods according to salt content ^7^. However, no regulations have been published that seek to reduce the sodium content in food preparations.

Determining the sodium content of foods prepared and sold outside the home would help to propose strategies for the management and control of noncommunicable diseases, particularly cardiovascular diseases, involving other actors in the culinary sector and local government authorities. For this reason, this study aimed to determine the sodium content of food sold in fast food establishments, traditional and typical food establishments, and street food outlets in Metropolitan Lima, as well as the proportion of foods that exceed the sodium content parameters of Peruvian law and English regulations.

**KEY MESSAGES**

Motivation for the study. The increasing consumption of away-from-home foods, which have high contents of critical nutrients associated with chronic diseases, makes it relevant to evaluate the sodium content of these preparations.

Main findings. The median sodium content in food consumed outside the home exceeded 400 mg/100 g, and 65% of the total preparations had high sodium content according to the parameters of Peruvian Law No. 30021.

Implications. The high sodium content in the evaluated foods, added to their frequent consumption, suggest a high intake of this nutrient in the population and the need to address this public health problem.

## MATERIALS AND METHODS

A cross-sectional study was conducted to evaluate the sodium content in frequently consumed away-from-home food in three areas of Metropolitan Lima, Peru. In February 2019, a group of experts was formed consisting of five local government officials, three nutritionists, a chef, five university professors, and a representative of a restaurant chain. These experts identified the away-from-home foods based on their experience in different fields of work related to the consumption, preparation, sale, study and control of food. Twenty types of food were selected, including those frequently consumed by the population and included in one of the three categories considered in this study. Beverages were excluded, since their sodium content is usually low. The categories were determined according to the establishment where the preparations were sold: preparations from fast food chains (n=6), preparations from street vendors (n=7) and preparations from traditional and typical food establishments (n=7) (Table 1) ^8^.

**Table 1:** Foods, ingredients and median sodium content (mg) per 100 g of the analyzed preparations.

Category	Preparation	Main ingredients	Median sodium content (mg)/100 g of the preparation (IQR)
Fast food	Hamburger	Bread, hamburger, oil	407.00 (111.78 - 536.40)
	French fries	Potatoes, vegetable oil, salt	375.58 (144.81 - 721.18)
	Pizza	Dough based on flour, salt and yeast; cheese, tomato sauce and English ham are added.	566.88 (461.00 - 675.62)
	Broaster chicken (fried chicken)	Chicken, oil, salt, flour for coating	531.88 (369.14 - 789.90)
	*Salchipapa* (Sausages and French fries)	Potatoes, sausage, oil and salt	321.42 (113.55 - 795.67)
	Wrap	Corn tortilla, fried chicken and fresh vegetables	623.57 (318.59 - 725.33)
Street food	*Anticucho*(Fried grilled beef heart)	Beef heart, *panca* chili, salt, pepper, cumin, garlic and vinegar	725.90 (418.82 - 908.23)
	Popcorn	Popcorn, vegetable oil and salt	240.84 (82.42 - 470.13)
	Hamburger	Yolk bread, hamburger and vegetable oil	583.95 (190.53 - 833.32)
	Egg sandwich	Bread, egg, vegetable oil and salt	359.81 (206.53 - 527.28)
	Avocado sandwich	Bread, avocado and salt	270.60 (200.82 - 368.17)
	Tortilla sandwich (Tortilla: mix of egg and sausage)	Bread, egg, sausage, vegetable oil and salt	479.27 (321.44 - 631.45)
	*Chicharrón*(Chopped and fried pork)	Pork, oil and salt	786.15 (419.77 - 1356.77)
Traditional and typical preparations	Chinese rice (Sauteed rice with chicken, egg, sausage and soy sauce)	Rice, chicken, egg, sausage, soy sauce, scallion, vegetable oil, garlic, ginger and salt.	662.40 (399.63 - 932.34)
	*Pollo a la brasa* (Charcoal-baked chicken))	Chicken, beer, vinegar, soy sauce, *panca* chili, garlic, oregano, and cumin	600.75 (393.58 - 849.24)
	*Ceviche*(Raw fish cooked with lemon)	Fish, lemon juice, onion, chili pepper and salt	633.47 (331.13 - 865.51)
	Sauteed noodles (Sauteed pasta with chicken and vegetables)	Noodles, chicken, vegetables, soy sauce, vegetable oil and salt	454.44 (375.56 - 542.41)
	Chicken broth	Wheat noodles, chicken, egg, potato, scallion, *cancha serrana* (fried corn with oil and salt) and salt.	273.25 (210.62 - 722.02)
	Tamales	Corn, vegetable shortening, yellow chili, chicken, olives, garlic	418.62 (369.64 - 528.14)
	*Papa a la huancaína* (Cooked potato accompanied by a cookie, milk and yellow chili sauce).)	Potato, yellow chili, cheese, milk, crackers, olives, oil and salt.	256.64 (74.25 - 342.00)

IQR: interquartile range.

The categorization of preparations was based on an Argentine study in order to define the types of food prepared outside of home ^8^. Fast food consists of culinary preparations of fast and systematic preparation, sold in cafeterias, take-away stores and chain restaurants, in some cases, transnational chains; street food preparations are preparations prepared and sold by street vendors; and traditional and typical preparations are usually prepared by hand in small or family businesses, sold in formal establishments, and are part of the country’s gastronomic heritage and consumed mostly by the local population ^8^.

**Figure 1:**
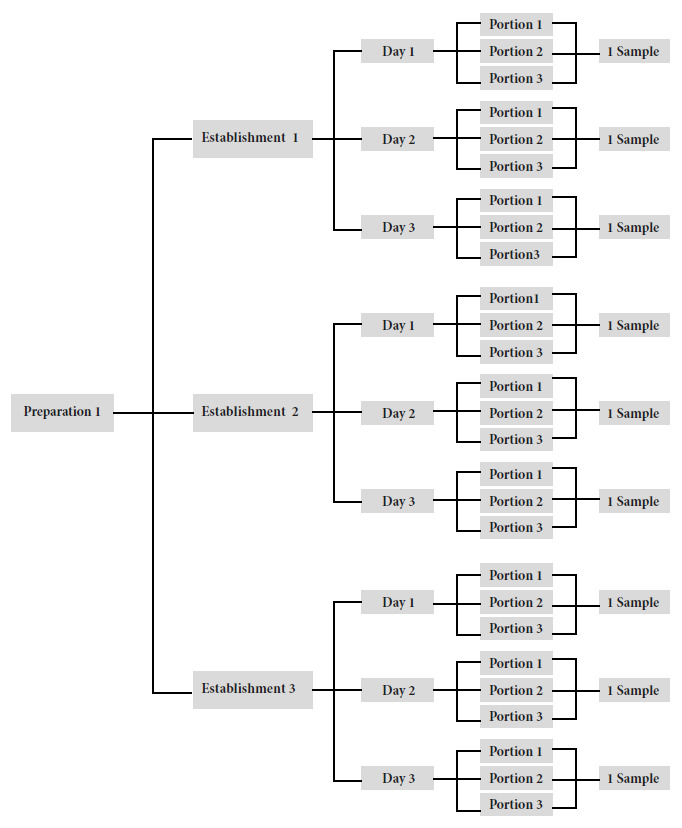
Sample collection for each food.

The study was conducted from March to July 2019, in six districts of the city of Metropolitan Lima of different socioeconomic levels: one in northern Lima (San Martin de Porres), four in central Lima (Cercado de Lima, San Miguel, Magdalena del Mar, Miraflores) and one in southern Lima (Villa María del Triunfo). These districts were selected because of the accessibility of the research team in terms of distance. Each type of food was collected in three different establishments, on three different dates, and three portions were collected on each date. It should be noted that some establishments offer more than one type of the preparations included in the study, so more than one type of preparation was purchased in these establishments. The three portions were mixed in the laboratory and a sample was obtained for each day of collection, resulting in three samples for each establishment and therefore nine samples for each preparation (Figure 1).

Foods were acquired without garnishes or accompaniments, and the research team also ensured that, on each collection date, each sample consisted of the same basic components; for example, the avocado sandwich always had to contain: bread, avocado and salt. All packaging and inedible parts were removed after acquiring each sample. Then each portion was weighed and photographed; each one had to have a minimum net weight of 100 g to ensure a sufficient amount of sample for chemical analysis. Each portion was registered with a unique code on a card, detailing the date and place of collection. Once coded, each of the 3 portions acquired on the same date and place were placed in an airtight bag and labeled with the corresponding code and then transferred to the laboratory in isothermal containers on the same day they were obtained.

The sodium content of each sample was obtained by atomic absorption spectroscopy (AACC Method 40-71) ^9^. Moisture content was previously determined under NON-116-SSA1(1994) ^10^^)^ and ash content under NMX-F607-NORMEX (2013) ^11^, which were developed by the ALS Global laboratory team, Peru. This process was standardized according to the Interlaboratory Rounds Program for Food Analysis (PRIDAA). The analysis of each sample was performed in duplicate, thus obtaining 18 values for each preparation.

In the absence of regulations or parameters for sodium content in culinary preparations, sodium content was compared with the parameters established in the second phase of implementation of Peruvian Law No. 30021, which were established to regulate beverages and processed and ultra-processed foods; considering foods with high sodium content as those with sodium values above 400 mg per 100 g ^6^. Additionally, as a further comparison, we used the levels stated in the UK Front of Packaged Food Nutrition Labeling Guide, which classifies solid packaged foods as low (≤120 mg/100 g), medium (> 120 mg and ≤600 mg/100 g) and high sodium content (> 600 mg/100 g) ^7^.

Continuous variables were described according to their distribution (median, interquartile ranges [IQR]), and visually by using violin diagrams. Categorical variables were grouped according to sodium content, indicating the percentages in each category. The statistical package Stata v.15 (Stata Corp., College Station, TX, US) was used.

## RESULTS

According to food categories, the median sodium content was 471.06 mg/100 g (IQR: 115.31 - 695.18) in fast food, 471.37 mg/100 g (IQR: 76.04 - 765.39) in traditional and typical preparations and 492.36 mg/100 g (IQR: 83.93 - 918.78) in street food, the median sodium content per preparation can be seen in Table 1.

**Figure 2:**
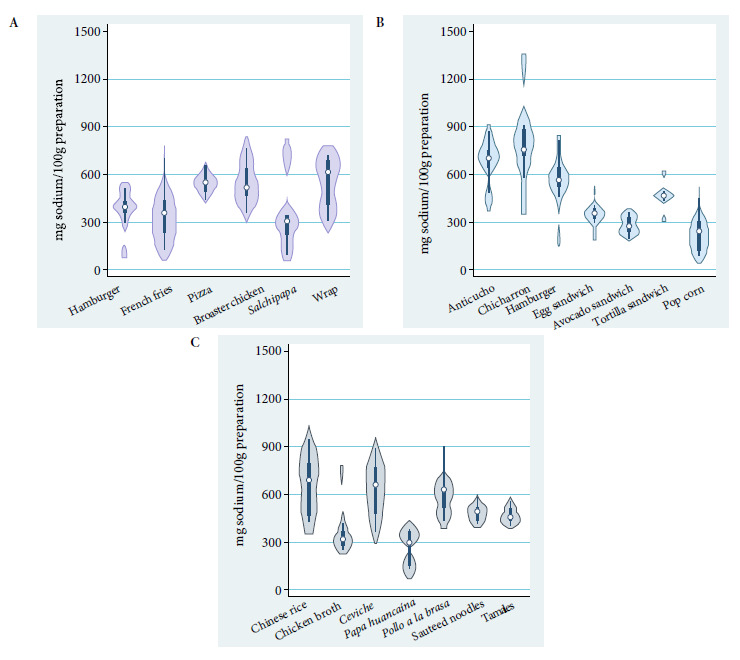
(a) Sodium content per 100 grams of fast food preparations. (b) Sodium content per 100 grams of street food preparations. (c) Sodium content per 100 grams of traditional and typical preparations.

The wrap was the preparation with the highest sodium content among fast foods, while “*salchipapa*” (fried sausages and French fries) had the lowest content. The preparation with the highest sodium content among traditional and typical preparations was “*arroz chaufa*” (Chinese rice), while the lowest was “*papa a la huancaína*” (Huancayo style potatoes). The preparation with the highest sodium content was “*chicharrón*” (fried pork) and the preparation with the lowest sodium content was popcorn. We found that the hamburger categorized as fast food had a lower sodium content than the one categorized as a street preparation (Figure 2).

**Figure 3:**
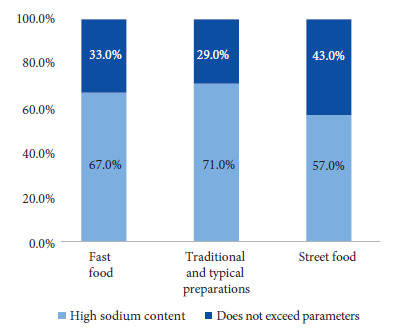
Proportion of away-from-home food with high sodium content according to the second phase of Peruvian Law No. 30021 (per 100 g of solid food).

We found that 65% (13/20) of the total preparations were classified as having high sodium content (>400 mg/100 g), according to the parameters of the second phase of the implementation of Peruvian Law No. 30021. The proportions of foods exceeding the levels established in the Peruvian Law, according to each category, were 71% (5/7) for traditional and typical preparations, 67% (4/6) for fast food, 57% (4/7) for street food preparations (Figure 3).

**Figure 4:**
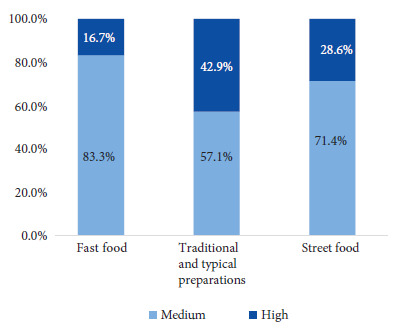
Proportion of away-from-home food with low, medium and high sodium content according to parameters for sodium content per 100 g of food established by the United Kingdom. No preparation had values considered as low.

According to the parameters established by the United Kingdom ^8^, 30% (6/20) of the preparations had high sodium content and 70% (14/20) had medium sodium content, while no preparation qualified as having low sodium content. Of the traditional and typical preparations, 42.9% (3/7) had high sodium content and 57.1% (4/7) had medium sodium content. High sodium content was found in 28.6% (2/7) of street foods and medium content in 71.4% (5/7). Finally, 16.7% (1/6) of fast foods had high sodium content and 83.3% (5/6) had medium content (Figure 4).

## DISCUSSION

The sodium content of away-from-home food has not been evaluated in most low- and middle-income countries, despite their high availability and frequent consumption. The present study sought to evaluate the sodium content in food sold in three areas of Metropolitan Lima. Our results show a high variability, since we found a wide range for each preparation. Many of these preparations, commonly consumed by the population of Metropolitan Lima, contain high levels of sodium according to the parameters established by Peruvian Law No. 30021 and the United Kingdom Guidelines.

It is important to consider that many of these foods are consumed in quantities that exceed 100 g, which has been the measure used to standardize and present the results. For example, a wrap can weigh a little more than 200 g, so the sodium content could be around 1500 mg per serving, equivalent to 75% of the WHO daily sodium intake recommendation ^1^. Therefore, in addition to the sodium levels described for amounts of 100 g that allow a comparison between the preparations, it is necessary to take into account the size of the portions to have a better approximation of their sodium contribution to the diet. A study in Argentina was found that the sodium content in the standard portion of chicken with salad from fast food establishments exceeded the WHO daily recommendation ^8^. A Mexican study reported that tacos can contribute almost 500 mg of sodium per day, equivalent to almost 25% of the recommendation ^12^.

Another variable that can increase the sodium intake of these foods is the frequency with which they are consumed. In Argentina, sodium contents similar to those found in this study (>500 mg of sodium/100 g of food) were found in the three food categories, although with different preparations. In addition, the frequency of food consumption outside the home was evaluated in this study, and the results showed a consumption between 4 to 6 times per week and sometimes more than once per day ^8^, so that the sodium intake from these foods may even be much higher than recommended due to the frequency of consumption.

The range of sodium content was wide among preparations in the same category, but also within the same preparation. This could be explained by the fact that each establishment may prepare food differently, and at the same time, the result suggests that it would be feasible for recipes to be changed to decrease the amount of sodium. This wide variability in sodium content has also been reported by other studies. For example, in Costa Rica, a study that analyzed preparations sold in fast food establishments found that the sodium content of the preparations ranged from less than 1000 mg/100 g to 2700 mg/100 g among restaurants offering chicken-based preparations ^13^. Coincidentally, in our study, Broaster chicken from fast food restaurants was found to be one of the preparations with a wider range of sodium content.

One of the main strengths of this study is that we used an objective methodology for determining sodium content, based on the proximal chemical analysis of foods and not through indirect techniques such as the declaration of nutritional composition. In addition, the preparations were repeatedly sampled on different dates at the selected points of sale, which made it possible to capture the sodium content of the foods more accurately. On the other hand, this study is novel, since there are few studies in Latin America that have evaluated the sodium content of foods sold on the street, recognizing that some of the preparations are part of the gastronomic identity of the country and are regularly consumed by the population.

One of the limitations of this study is that we selected only twenty of the great variety of existing preparations, and these were not representative of all of Metropolitan Lima, due to the lack of an official and publicly accessible registry of all the establishments that sell food in Metropolitan Lima, including street stalls. Therefore, we sought to evaluate greater diversity of preparation methods and carried out the sampling in districts with different socioeconomic levels. Another limitation was related to the fact that the most consumed foods were selected by a panel of experts; in addition, the categorization of the types of food was based on a previous study from another country in the region, due to the absence of objective definitions and the lack of information at the national or local level on the most consumed away-from-home foods. Consequently, it is necessary to consider that our results are valid for the studied sample and that it is advisable to standardize the classification of foods in order to carry out comparable studies. Finally, in the absence of established parameters for categorizing sodium content in foods prepared away from home, we used the current national and international standards for processed and ultra-processed products as reference. Although the parameters we used have not been properly established to analyze this type of foods, they serve as reference to categorize and compare preparations according to sodium content in the absence of specific parameters. A previous study in the region faced the same issue ^8^; therefore, it should not be assumed that these parameters are necessarily optimal, so future studies must establish proper cut-off points for these foods, since they are increasingly consumed.

In conclusion, our results show that the median sodium content in each food category exceeded 400 mg of sodium per 100 g. In addition, most of the preparations were considered to have high sodium content according to the parameters of Peruvian Law No. 30021. These results are an alert for the country’s health authorities and the actors directly involved in the preparation and sale of food. It is well known that the consumption of away-from-home food is a common practice in Peru, as well as in Latin America ^5^; therefore, it is essential to develop interventions focused on prevention and changing habits, preferably participatory strategies, designed in conjunction with key actors, to prevent these measures from being rejected by the population ^14^^,^^15^. The implementation of policies and programs focused on reducing the sodium content of away-from-home foods within the framework of the National Multisectoral Health Policy that seeks to improve healthy habits, behaviors and lifestyles of Peruvians ^16^ can produce health benefits. These initiatives should be based on current evidence ^17^, as well as in future research that should assess a greater variety of foods at the national level, in order to generate useful data for the authorities and thus expand knowledge on the frequency of food consumption outside the home, as the trend towards greater consumption of these foods is imminent.
