# Supplementary material for: Sodium content in commonly consumed away-from-home food in three areas of Metropolitan Lima, Peru
Source: Rev Peru Med Exp Salud Publica. 2023 Sep 27;40(3):340–7. doi: 10.17843/rpmesp.2023.403.12939 (PMC10953669; doi:10.17843/rpmesp.2023.403.12939)
Supplement: Translation

# Translation: Contenido de sodio en preparaciones de consumo frecuente fuera del hogar en tres zonas de Lima Metropolitana, Perú

**Authors:** Mayra Meza-Hernández, Rafael Durán-Galdo, Daniella Torres-Schiaffino, Lorena Saavedra-Garcia

**Affiliations:** 1 CRONICAS Centro de excelencia en enfermedades crónicas, Universidad Peruana Cayetano Heredia. Lima, Perú.

**Keywords:** Alimentos Vendidos en la Vía Pública, Comida Rápida, Sodio, Perú

## Abstract

El objetivo fue caracterizar el contenido de sodio en preparaciones de consumo frecuente fuera del hogar en tres zonas de Lima Metropolitana. Se realizó un estudio transversal para el cual se identificaron veinte preparaciones de consumo frecuente según el lugar de expendio. La determinación del contenido de sodio se obtuvo mediante espectroscopía de absorción atómica en preparaciones recolectadas en el 2019. La mediana del contenido de sodio en productos de venta ambulatoria fue 492,36 mg/100 g (RIQ: 83,93 - 918,78); 471,37 mg/100 g, en preparaciones tradicionales y típicas (RIQ: 76,04 - 765,39); y 471,06 mg/100 g (RIQ: 115,31 - 695,18), en comidas rápidas. El 65% de las preparaciones fueron consideradas altas en sodio según los parámetros peruanos, mientras que el 30% de las preparaciones también presentaron un alto contenido, de acuerdo con los parámetros del Reino Unido. La mayoría de las preparaciones expendidas y consumidas fuera del hogar presentan un elevado contenido de sodio. Es esencial involucrar a todos los actores que participan en la preparación de alimentos para el consumo fuera del hogar, con el fin de sensibilizarlos e incluirlos en la promoción de políticas enfocadas en la reducción del consumo de sodio.

## INTRODUCCIÓN

En muchos países, el consumo de sodio en la dieta supera la recomendación de la Organización Mundial de la Salud (OMS) de 2000 mg de sodio diarios, equivalente a 5000 mg de sal ^1^. Un ejemplo de esto se encuentra en Latinoamérica, donde el promedio del consumo de sodio es de 4130 mg al día ^2^. Este elevado consumo de sodio es uno de los factores de riesgo para el desarrollo de enfermedad cardiovascular, insuficiencia renal y muerte prematura ^1^.

En países desarrollados, tanto los alimentos procesados y ultraprocesados, así como los alimentos preparados fuera del hogar, representan la principal fuente de sodio en la dieta ^3^. Por el contrario, en los países en desarrollo las principales fuentes de sodio se circunscriben a la sal añadida en las preparaciones culinarias hechas en el hogar ^4^. Sin embargo, como parte de la transición nutricional, el consumo de alimentos preparados fuera del hogar está incrementando. Por lo tanto, la preparación de alimentos de venta de ambulatoria cobra actualmente gran interés debido a que su consumo es muy frecuente y parte de la cultura gastronómica de estos países ^5^.

En respuesta al incremento del consumo de sodio y sus consecuencias en la salud se han desarrollado diversas estrategias. Por ejemplo, en Perú se promulgó la Ley N.º 30021, «Ley de promoción de la alimentación saludable para niños, niñas y adolescentes» la cual establece parámetros para determinar el contenido alto de nutrientes como el sodio en alimentos procesados y ultraprocesados ^6^. Así también, Reino Unido ha establecido parámetros para clasificar los alimentos procesados y ultraprocesados de acuerdo con el contenido de sal ^7^. Sin embargo, no se han publicado reglamentaciones que busquen reducir el contenido de sodio en las preparaciones culinarias.

Conocer el contenido de sodio de los alimentos preparados y expendidos fuera del hogar ayudaría a plantear estrategias para el manejo y control de las enfermedades no transmisibles, específicamente las enfermedades cardiovasculares, involucrando a otros actores del sector culinario y autoridades de los gobiernos locales. Por tal motivo, el objetivo de este estudio fue determinar el contenido de sodio y en preparaciones expendidas en establecimientos de comida rápida, de comida tradicional y típica, y de venta ambulatoria en Lima Metropolitana, así como la proporción de preparaciones que superan los parámetros para el contenido de sodio de la Ley peruana y la normativa inglesa.

**MENSAJES CLAVE**

Motivación para el estudio. El incremento del consumo de alimentos fuera del hogar, altos en nutrientes críticos asociados a enfermedades crónicas, hace relevante evaluar el contenido de sodio de estas preparaciones.

Principales hallazgos. La mediana del contenido de sodio en las preparaciones consumidas fuera del hogar supera los 400 mg/100 g, además el 65% del total de preparaciones tuvieron alto contenido de sodio según los parámetros de la Ley peruana N° 30021.

Implicancias. El alto contenido de sodio en las preparaciones evaluadas, sumado a su frecuente consumo, sugieren una elevada ingesta de este nutriente en la población y la necesidad de abordar esta problemática de salud pública.

## MATERIALES Y MÉTODOS

Se realizó un estudio transversal para evaluar el contenido de sodio presente en las preparaciones de consumo frecuente fuera del hogar en tres zonas de Lima Metropolitana, Perú. En febrero del 2019, se formó un grupo de expertos conformado por cinco funcionarios de gobiernos locales, tres nutricionistas, un chef, cinco docentes universitarios y un representante de una cadena de restaurantes. Estos expertos identificaron las preparaciones frecuentemente consumidas fuera del hogar basándose en la experiencia adquirida en sus distintos ámbitos laborales relacionados al consumo, preparación, venta, estudio y fiscalización de alimentos. Se seleccionaron 20 tipos de preparaciones, se incluyeron aquellas de consumo frecuente por la población y que se encuentran incluidas en alguna de las tres categorías de alimentos considerados en este estudio. Se excluyeron las bebidas, ya que su contenido de sodio es usualmente bajo. Las categorías se determinaron según el establecimiento en donde se expenden las preparaciones: preparaciones de cadenas de comida rápida (n=6), preparaciones de venta ambulatoria (n=7) y preparaciones de establecimientos de venta de comida tradicional y típica (n=7) (Tabla 1) ^8^.

**Tabla 1:** Preparaciones, ingredientes y mediana del contenido de sodio (mg) por cada 100 g de preparación analizada.

Categoría	Preparación	Ingredientes principales	Mediana del contenido de sodio (mg)/100 g de preparación (RIC)
Comida rápida	Pan con hamburguesa	Pan, hamburguesa, aceite	407,00 (111,78 - 536,40)
	Papas fritas	Papas, aceite vegetal, sal	375,58 (144,81 - 721,18)
	Pizza	Masa en base a harina, sal y levadura; los añadidos son queso, salsa de tomate y jamón inglés.	566,88 (461,00 - 675,62)
	Pollo Broaster (*Pollo frito*)	Pollo, aceite, sal, harina para empanizar	531,88 (369,14 - 789,90)
	Salchipapa (*Papas fritas con salchichas fritas*)	Papas, salchichas, aceite y sal	321,42 (113,55 - 795,67)
	Wrap	Tortilla de maíz, pollo frito y verduras frescas	623,57 (318,59 - 725,33)
Preparaciones de venta ambulatoria	Anticucho (*Trozos de corazón de res fritos a la parrilla*)	Corazón de res, ají panca, sal, pimienta, comino, ajos y vinagre	725,90 (418,82 - 908,23)
	Pop corn	Maíz pop corn, aceite vegetal y sal	240,84 (82,42 - 470,13)
	Pan con hamburguesa	Pan de yema, hamburguesa y aceite vegetal	583,95 (190,53 - 833,32)
	Pan con huevo frito	Pan, huevo, aceite vegetal y sal	359,81 (206,53 - 527,28)
	Pan con palta	Pan, palta (aguacate) y sal	270,60 (200,82 - 368,17)
	Pan con tortilla (*Tortilla: mezcla de huevo y salchichas*)	Pan, huevo, salchicha, aceite vegetal y sal	479,27 (321,44 - 631,45)
	Chicharrón (*Carne de cerdo troceada y frita*)	Carne de cerdo, aceite y sal	786,15 (419,77 - 1356,77)
Preparaciones tradicionales y típicas	Arroz chaufa (*Arroz salteado con pollo, huevo, salchicha y salsa de soja*)	Arroz, pollo, huevo, salchicha, salsa de soja, cebolla china (cebollín), aceite vegetal, ajo, kion (jengibre) y sal.	662,40 (399,63 - 932,34)
	Pollo a la brasa (*Pollo horneado con carbón*)	Pollo, cerveza, vinagre, sillao, ají panca, ajo, comino, orégano y comino	600,75 (393,58 - 849,24)
	Ceviche (*Pescado crudo cocido con limón*)	Pescado, jugo de limón, cebolla, ají y sal	633,47 (331,13 - 865,51)
	Tallarín saltado (*Pasta salteada con pollo y verduras*)	Pasta (tallarín), pollo, verduras, salsa de soja, aceite vegetal y sal	454,44 (375,56 - 542,41)
	Caldo de gallina	Fideos de trigo, gallina, huevo, papa, cebolla china (cebollín), cancha serrana (maíz frito con aceite y sal) y sal.	273,25 (210,62 - 722,02)
	Tamales	Maíz, manteca vegetal, ají amarillo, pollo, aceituna, ajo	418,62 (369,64 - 528,14)
	Papa a la huancaína (*Papa cocida acompañada de salsa a base de galleta, leche y ají amarillo*)	Papa, ají amarillo, queso, leche, galleta, aceituna, aceite y sal.	256,64 (74,25 - 342,00)

RIC: rango intercuartílico

La categorización se basó en un estudio argentino para definir los tipos de alimentos preparados fuera del hogar ^8^. La *comida rápida* consiste en preparaciones culinarias de ágil y sistemática elaboración, vendidos en cafeterías, tiendas para llevar y cadenas de restaurantes, en algunos casos, cadenas de ámbito transnacional; las *preparaciones de venta ambulatoria* son preparaciones elaboradas y expendidas en la calle por vendedores ambulantes; y las *preparaciones tradicionales y típicas* son usualmente elaboradas de forma manual en negocios pequeños o negocios familiares, expendidas en establecimientos formales, además de formar parte de la riqueza gastronómica del país y consumidas mayormente por la población local ^8^.

El estudio se desarrolló entre los meses de marzo a julio del año 2019, en seis distritos de la ciudad de Lima Metropolitana de diferentes niveles socioeconómicos: uno en la zona norte (San Martin de Porres), cuatro en la zona centro (Cercado de Lima, San Miguel, Magdalena del Mar, Miraflores) y uno en la zona sur (Villa María del Triunfo). Estos distritos fueron seleccionados por la accesibilidad del equipo de investigación en términos de distancia. Cada tipo de preparación se recolectó en tres establecimientos diferentes, en tres fechas distintas y en cada fecha se recolectaron tres porciones. Cabe resaltar que algunos establecimientos ofrecen más de un tipo de las preparaciones incluidas en el estudio, entonces en dichos establecimientos se adquirió más de un tipo de preparación. Las tres porciones recolectadas se mezclaron en el laboratorio y se obtuvo una muestra por cada día de recolección, resultando tres muestras por cada establecimiento y por ende nueve muestras por cada preparación (Figura 1).

**Figura 1:** Recolección de muestras por cada preparación.

Las preparaciones fueron adquiridas sin guarniciones ni acompañamientos, así también el equipo de investigación se aseguró que, en cada fecha de recolección, cada muestra recogida este conformada de los mismos componentes base, por ejemplo, el pan con palta siempre debía llevar: pan, palta y sal. Luego de adquirida cada muestra se retiró todo tipo de empaque y partes no comestibles, se procedió a pesar y fotografiar cada porción, la cual debía tener un peso neto mínimo de 100 g para asegurar una cantidad de muestra suficiente para los análisis químicos. Cada porción se registró con un código único en una ficha, detallando la fecha y el lugar de recolección. Una vez codificadas, cada una de las 3 porciones adquiridas en la misma fecha y lugar fueron colocadas en una bolsa hermética y rotuladas con el código correspondiente para luego ser trasladada al laboratorio en contenedores isotérmicos el mismo día que fueron adquiridas.

El contenido de sodio en las muestras se obtuvo mediante espectroscopía de absorción atómica (Método AACC 40-71) ^9^. Previamente se determinó el contenido de humedad bajo la normativa NON-116-SSA1(1994) ^10^ y el de cenizas bajo la normativa NMX-F607-NORMEX (2013) ^11^, los cuales fueron desarrollados por el equipo del laboratorio ALS Global, Perú. Este proceso fue estandarizado según el Programa de Rondas Interlaboratorio de Análisis de Alimentos (PRIDAA). El análisis de cada muestra adquirida se realizó por duplicado, obteniendo así 18 valores por cada preparación.

Ante la falta de regulaciones o parámetros para el contenido de sodio en preparaciones culinarias, se comparó el contenido de sodio con los parámetros establecidos en la segunda fase de implementación de Ley Peruana N° 30021, los cuales se establecieron para regular las bebidas y alimentos procesados y ultraprocesados; considerando como alto contenido de sodio cuando el alimento supera los 400 mg por cada 100 g ^6^. Adicionalmente, como comparación adicional, se usaron los niveles indicados en la Guía de Etiquetado Nutricional Frontal de Alimentos Envasados del Reino Unido, la misma que clasifica a los alimentos sólidos envasados como bajo (≤120 mg/100 g), medio (> 120 mg y ≤600 mg/100 g) y alto (> 600 mg/100 g) ^7^.

Las variables continuas fueron descritas según su distribución (mediana, rangos intercuartiles [RIC]) y de manera visual a través de diagramas de violín. Las variables categóricas fueron agrupadas según el contenido de sodio, indicando los porcentajes en cada categoría. Se utilizó el paquete estadístico Stata v.15 (Stata Corp., College Station, TX, US).

## RESULTADOS

Según las categorías de alimentos, la mediana del contenido de sodio fue 471,06 mg/100 g (RIC: 115,31 - 695,18) en la comida rápida, 471,37 mg/100 g (RIQ: 76,04 - 765,39) en las preparaciones tradicionales y típicas y 492,36 mg/100 g (RIQ: 83,93 - 918,78) en las de venta ambulatoria, la mediana del contenido de sodio por cada preparación se puede observar en la Tabla 1.

En el caso de las comidas rápidas, el *wrap* fue la preparación con mayor contenido de sodio mientras que la salchipapa tuvo el menor contenido. La preparación con mayor contenido de sodio entre las preparaciones tradicionales y típicas fue el arroz chaufa, mientras que la de menor contenido fue la papa a la huancaína. Con relación a las preparaciones de venta ambulatoria, la preparación con mayor contenido de sodio fue el chicharrón (carne de cerdo frita) y la preparación con menor contenido fue el *pop corn*. Se encontró que la hamburguesa categorizada como comida rápida presenta menor contenido de sodio que la que se categoriza como preparación de venta ambulatoria (Figura 2).

**Figura 2:** (a) Contenido de sodio por cada 100 gramos de preparaciones de establecimientos de comida rápida. (b) Contenido de sodio por cada 100 gramos de preparaciones de venta ambulatoria. (c) Contenido de sodio por cada 100 gramos de preparaciones tradicionales y típicas.

Con relación a la comparación con los puntos de corte establecidos en la Ley Peruana Nº 30021, el 65% (13/20) del total de preparaciones fue clasificada como altas en sodio (400 mg/100 g), de acuerdo con los parámetros de la segunda fase de la implementación de la Ley. Las proporciones de preparaciones que superan los niveles establecidos en la Ley Peruana, según cada categoría, fueron 71% (5/7) para las preparaciones tradicionales y típicas, 67% (4/6) para la comida rápida, 57% (4/7) para las preparaciones de venta ambulatoria (Figura 3).

**Figura 3:** Proporción de preparaciones fuera del hogar Alto en sodio según el parámetro del contenido de sodio, por cada 100 g de alimentos sólidos, establecido en la segunda fase de la Ley Peruana N° 30021.

Respecto a los parámetros establecidos por el Reino Unido ^8^, el 30% (6/20) de las preparaciones tuvo alto contenido de sodio y el 70% (14/20) mediano contenido de sodio, mientras que ninguna preparación calificó como bajo contenido de sodio. El 42,9% (3/7) de las preparaciones tradicionales y típicas presentó alto contenido de sodio y el 57,1% (4/7) tuvo contenido medio. El 28,6% (2/7) de preparaciones de venta ambulatoria presentó alto contenido de sodio y el 71,4% (5/7) tuvo contenido medio. Por último, el 16,7% (1/6) de comida rápida tuvo alto contenido de sodio y el 83,3% (5/6) tuvo contenido medio (Figura 4).

**Figura 4:** Proporción de preparaciones fuera del hogar con bajo, medio y alto contenido de sodio según parámetros para el contenido de sodio por cada 100 g de alimento establecido por el Reino Unido. Ninguna preparación presentó valores considerados como nivel bajo.

## DISCUSIÓN

El contenido de sodio en los alimentos preparados fuera del hogar no ha sido evaluado en la mayoría de los países de renta baja y media, a pesar de su alta disponibilidad y consumo frecuente. El presente estudio buscó evaluar el contenido de este mineral en preparaciones expendidas en tres zonas de Lima Metropolitana. Nuestros resultados señalan una alta variabilidad pues se encontró gran amplitud en los rangos de cada preparación. Muchas de estas preparaciones, consumidas comúnmente por la población de Lima Metropolitana contienen altos niveles de sodio según los parámetros establecidos por la Ley peruana N° 30021 y las Guías del Reino Unido.

Es relevante considerar que muchos de estos alimentos se consumen en cantidades que superan los 100 g, que ha sido la medida para estandarizar y presentar los resultados. Por ejemplo, un *wrap* puede pesar un poco más de 200 g, con lo cual el contenido de sodio podría estar alrededor de los 1500 mg por porción, equivalente a 75% de la recomendación diaria de ingesta de sodio de la OMS ^1^. Por ello, además de los niveles de sodio descritos para cantidades de 100 g que permite una comparación entre las preparaciones estudiadas, es necesario tomar en cuenta el tamaño de las porciones usualmente consumidas para tener una mejor aproximación de su aporte de sodio a la dieta. En Argentina, se encontró que el contenido de sodio en la porción estándar del pollo con ensalada provenientes de establecimientos de comida rápida superó la recomendación diaria de la OMS ^8^. En México, se ha reportado que los tacos pueden aportar casi 500 mg de sodio por día, un equivalente a casi el 25% de la recomendación ^12^.

Otra variable que puede incrementar el aporte de sodio de estos alimentos es la frecuencia con la que son consumidos. En Argentina, se encontraron contenidos de sodio similares a los encontrados en este estudio (>500 mg de sodio/100 g de alimento) en las tres categorías de preparaciones evaluadas, aunque con preparaciones distintas. Adicionalmente, en dicho estudio se evaluó la frecuencia de consumo de alimentos fuera del hogar, y los resultados mostraron un consumo de entre 4 a 6 veces por semana y en ocasiones más de una vez por día ^8^, por lo que el aporte de sodio proveniente de estos alimentos puede ser incluso muy superior a lo recomendado debido a la frecuencia de consumo.

El rango del contenido de sodio fue amplio entre las preparaciones de una misma categoría, pero también dentro de una misma preparación. Esto se podría explicar porque cada establecimiento puede tener un modo de preparación distinto, y a la vez, el resultado sugiere que sería factible que las recetas se reformulen para disminuir la cantidad de sodio. Esta amplia variabilidad en el contenido de sodio también ha sido reportada en otros estudios. Por ejemplo, en Costa Rica, una investigación donde se analizaron preparaciones expendidas en establecimientos de comida rápida encontró que entre los restaurantes que ofrecen preparaciones a base de pollo, el contenido de sodio en las preparaciones fluctúa desde menos de 1000 mg/100 g hasta 2700 mg/100 g ^13^. Coincidentemente, en el presente estudio se encontró que el pollo broaster de restaurantes de comida rápida es una de las preparaciones con un rango más amplio de contenido de sodio.

Una de las principales fortalezas del estudio es que la metodología para determinar el contenido de sodio fue objetiva, a partir del análisis químico proximal de alimentos y no mediante técnicas indirectas como la declaración de la composición nutricional. Adicionalmente se muestrearon repetidamente las preparaciones en fechas distintas en los puntos de venta seleccionados, lo cual permitió capturar con mayor precisión el contenido de sodio de los alimentos. Por otro lado, este estudio resulta novedoso, pues son pocas investigaciones en Latinoamérica que han evaluado el contenido de sodio en alimentos expendidos de forma ambulatoria, reconociendo que algunas de las preparaciones estudiadas forman parte de la identidad gastronómica y son parte del consumo habitual de gran parte de la población.

Dentro de las limitaciones de este estudio debemos señalar que solo se seleccionaron veinte de la gran variedad de preparaciones existentes y estas no fueron representativas de toda Lima Metropolitana, esto ante la falta de un registro oficial y de acceso público de todos los establecimientos que expenden alimentos en Lima Metropolitana, incluyendo los puestos de venta ambulatoria. Por ello, buscamos mayor diversidad en el método de preparación y llevamos a cabo el muestreo en distritos con diferente nivel socioeconómico. Consideramos que otra limitación estuvo relacionada a la selección de las preparaciones más consumidas, debido a que estas fueron seleccionadas por un panel de expertos; además, la categorización de los tipos de comida se basó en un estudio previo de otro país de la región, debido a la ausencia de definiciones objetivas y a la falta de información a nivel nacional o local sobre las preparaciones de venta fuera del hogar más consumidas por la población. En consecuencia, es necesario considerar que los resultados son válidos para la muestra estudiada y que es recomendable estandarizar la clasificación de los alimentos para generar estudios comparables. Finalmente, ante la no existencia de parámetros establecidos para categorizar el contenido de sodio dentro de los alimentos preparados fuera del hogar, se usó como referencia la normativa vigente nacional e internacional para productos procesados y ultraprocesados. Si bien los parámetros usados no han sido establecidos para el tipo de alimentos analizados en este estudio, sirven como un referente para categorizar y comparar las preparaciones según contenido de sodio ante la ausencia de parámetros específicos, como también sucedió en otro estudio de la región ^8^, y no debe asumirse que son necesariamente los óptimos, por lo que sería ideal que se realicen estudios para establecer los puntos de corte para estos alimentos, ya que son cada vez más consumidos.

En conclusión, en el presente estudio encontramos que la mediana del contenido de sodio en cada categoría de alimentos superó los 400 mg de sodio por cada 100 g de alimento. Además, la mayoría de las preparaciones analizadas son consideradas altas en sodio según los parámetros de la Ley peruana N° 30021. Estos resultados evidencian una alerta para las autoridades sanitarias del país y los actores directamente involucrados con la preparación y expendio de preparaciones de alimentos. Es conocido que el consumo de alimentos fuera del hogar es una práctica habitual que forma parte de la cultura peruana, así como en América Latina ^5^; por ello, es fundamental desarrollar intervenciones enfocadas en la prevención y el cambio de hábitos, de preferencia estrategias participativas, diseñadas en conjunto con los actores clave, para evitar que estas medidas generen rechazo en la población ^14^^,^^15^. En el marco de la Política Nacional Multisectorial de Salud que busca mejorar hábitos, conductas y estilos de vida saludables de los peruanos ^16^, la implementación de políticas y programas enfocados en la reducción del contenido de sodio de alimentos expendidos fuera del hogar pueden generar beneficios en salud. Estas iniciativas deben surgir en base a la evidencia actual ^17^ y futuras investigaciones que consideren una mayor variedad de preparaciones a nivel nacional, para generar datos útiles a las autoridades y así ampliar el conocimiento sobre la frecuencia de consumo de alimentos fuera del hogar pues es inminente la tendencia a un mayor consumo de estos.
